# Dissolved Gases and Ice Fracturing During the Freezing of a Multicellular Organism: Lessons from Tardigrades

**DOI:** 10.1089/biores.2015.0008

**Published:** 2015-04-01

**Authors:** Gunther Kletetschka, Jolana Hruba

**Affiliations:** ^1^ Faculty of Science, Charles University in Prague, Prague, Czech Republic.; ^2^ Institute of Geology, Czech Academy of Sciences, v.v.i., Prague, Czech Republic.; ^3^ Lawrence Berkeley National Laboratory, Berkeley, California.

**Keywords:** cryopreservation, cryptobiosis, DNA damage, extracellular damage, survival

## Abstract

Three issues are critical for successful cryopreservation of multicellular material: gases dissolved in liquid, thermal conductivity of the tissue, and localization of microstructures. Here we show that heat distribution is controlled by the gas amount dissolved in liquids and that when changing the liquid into solid, the dissolved gases either form bubbles due to the absence of space in the lattice of solids and/or are migrated toward the concentrated salt and sugar solution at the cost of amount of heat required to be removed to complete a solid-state transition. These factors affect the heat distribution in the organs to be cryopreserved. We show that the gas concentration issue controls fracturing of ice when freezing. There are volumetric changes not only when changing the liquid into solid (volume increases) but also reduction of the volume when reaching lower temperatures (volume decreases). We discuss these issues parallel with observations of the cryosurvivability of multicellular organisms, tardigrades, and discuss their analogy for cryopreservation of large organs.

## Introduction

In this work, we set the working hypothesis stating that the gas concentration in the cryopreserved fluid has a critical role both in fracture development in the solid state and in the uniformity of heat removal.

## Gases Dissolved in Fluids

The gas content in cryopreserved material is limited by the gas solubility in a tissue containing fluid.^1,2^ The solubility of gases increases with both the gas pressure increase and temperature decrease.^3^ When the fluid converts to a solid material, the dissolved gases, which are too large to fit into the lattice of ice, migrate from the solution and are redistributed at the solid–water interface. As a result, during this process, the fluid along the interface should be enriched with gas.^1,3,4^ As freezing progresses, the concentration of dissolved gases surpasses a critical value, the water-containing fluid at the interface becomes supersaturated, and the gas bubbles may nucleate and grow to a visible size either along the interface and/or be trapped within the advancing solid.^3,5–7^

The gas concentration in body fluid has its role when cryopreserving organs for transplantation. It is critical to use Boutron's semi-empirical crystallization theory along with the microscopy method, to quantify gas content and ice in thin films of vitrification solutions.^8,9^ Dissolved gas in fluids was shown to play a role when studying the effect of cryopreservation of mouse embryo and isolated hepatocytes.^10,11^

In the context of cryopreservation, the variation of gas content changes homogeneity of the heat flow at the time of cooling. When the gas content decreases, it limits the number of sites where the ice/bubble can nucleate. Because the locations, where the ice/bubbles nucleate, have contrasting heat flow properties,^12^ these attenuate the heat flow. Therefore, the heat flow with lowered gas content becomes more uniform and stable compared with the fluid with increased gas content. Related heat flow changes can be observed, for instance, where initially warm water containing less dissolved gas freezes before colder water containing more of the dissolved gases. This phenomenon, also known as the Mpemba effect,^13–17^ is caused by various cooling conditions. Evaporative cooling, dissolved gases, convection, the surrounding environment, and supercooling are parameters that control the magnitude of the Mpemba effect.^14^

A more complex condition is the temperature distribution of the fluid at the time of cooling. As the fluid cools, it develops convection currents, the temperature becomes nonuniform, and the fluid is no longer characterized by simple temperature gradients.^14^ Convection currents cause the initially warmer fluid to have a higher rate of heat transfer from its top surface.^18^ The heat transfer efficiency is also influenced by the shape and dimension of the fluid volume geometry.^15^

Warm and cold fluids differ in their equilibrium gas content and this is what controls the heat conduction properties of the water-rich fluid.^14,17^ Experiments indicate that the Mpemba effect occurs in water-rich fluid with removed dissolved gases, such as dissolved CO_2_.^17,19^ Reports on the onset of the Mpemba effect suggest that this is due to a combination of the following three parameters. First parameter is a convective transport of heat in fluid by the gases dissolved in the fluid. The second parameter is an increase in the enthalpy of freezing of fluid by the dissolved gases. Temperature measurements showed that initially warmer water begins to freeze before the cold water due to supercooling.^13,20^ In this case, the initially colder water supercools to a lower temperature before it started to freeze. The third parameter is a decrease in the heat conduction of the solid due to bubbles of incorporated gases.^4,17^

## Fracturing of Solids When Lowering the Temperature

**FIG. 1.**
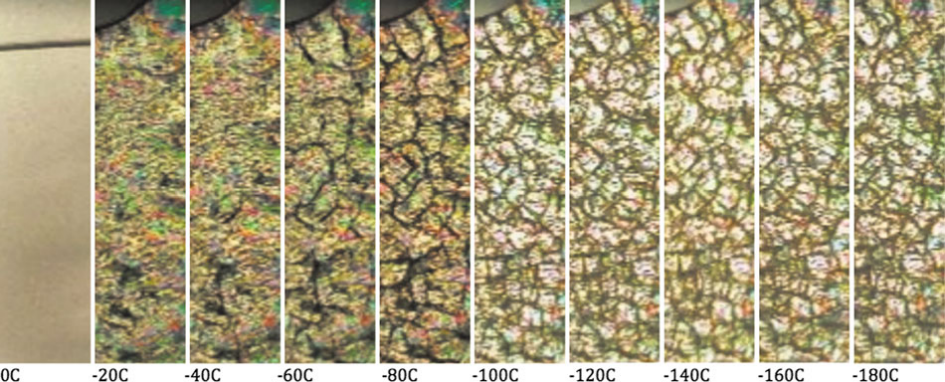
Slab of water was cooled down toward the liquid nitrogen temperature. Left image shows transmission of light through the 0.5-mm-thick slab (1×4.5 cm) of deionized water. Following panels show development of ice fractures in cross-polarized light. Decreasing temperature value is shown under each image. Cooling rate for this process was about 1 K/min.

Mixtures of fluids and solids produce volumetric changes with temperature. When a fluid is transferred into the solid that contains mostly water ice, there is a positive change in volume (volume of solids increases). When the temperature of this solid is further reduced, there is more than one generation of fractures. This is illustrated by an experimental observation^21^ and shown in Figure 1. The reason for fractures in ice is due to the change of lattice spacing when the temperature changes. At first, when the liquid water transfers into the ice water, the volume increases by about 10% compared with the volume of the liquid. However, with further temperature reduction, the lattice dimension of ice decreases and creates accumulation of stress conditions within the overall ice structure. As the stress increases, the ice fractures to reduce the accumulated stress. Fractures are located through the places with local maxima of gas and salt concentrations. The fractures continue to develop as the solidified samples cool to a lower temperature. Once the material achieves a temperature of 50 K, the lattice reduction rate slows down and fracture frequency stops.^22^ Thermal stress-induced fractures render cryopreserved frozen tissue unusable for transplantation.^23^ Rewarming of such frozen tissue with fractures results in bleeding and medical complications during the cryosurgery.^23^

To possibly combat intense fracturing, inert helium gas has recently been used as an agent to remove the heat from an organ interior during freezing. Steve van Sickle presented in the SENS 6 conference a dataset(s) that showed efficient heat exchange using the precooled helium gas as a heat transfer medium,^24^ removing the heat from the organs while flowing through the circulatory system of the kidney. The helium gas flow method was claiming potential for lowering the tissue damage during cryopreservation by vitrification applicable for organ transplants.^25–28^ Two limitations to vitrification are thermomechanical fracturing and the biochemical toxicity of vitrification solutions. Helium application was shown to eliminate fracturing and reduce the effective toxicity of vitrifiable cryoprotectants in vitrified swine kidneys, as well as increased cooling rates from 0°C to −100°C, reducing exposure time to cryoprotectant solutions. However, the helium gas may still form solutions inside the cryoprotectant. This may lead to compromising the uniformity of the heat removal from the organ and preferential fracture development through the nonuniform solids.

## Hibernation of Multicellular Organisms, Lessons from Other Life Forms

Invertebrates such as nematodes and tardigrades, selected bacteria, yeasts, and plants share an important trait. This is their ability to survive in an environment almost completely devoid of water. For decades the dry organisms are able to maintain anhydrobiosis without apparent damage.^29–31^ Following hydration, they resume their active life.^32^

Processes involving desiccation initiate substantial damage to both proteins and membranes, triggering a wave of apoptosis followed by death of the entire individual organism.^31^ A desiccated organism cannot use any reparation mechanisms to damaged macromolecules or proteins, since their metabolism is arrested with no liquid water available. This way, significant DNA damage can accumulate to exceed a certain level that prevents the cells' ability to repair.^33,34^

There are numerous anhydrobiotic organisms that acquired unique mechanisms, which allow them to escape from damage or to successfully repair damage that arises, while surviving extreme dehydration in their cryptobiotic form. This effect goes as far as survival in space conditions^35^ and exposure to temperatures ranging from absolute zero to temperatures near 373 K.^36^ Among the proposed survival mechanisms is an accumulation of compatible osmolites, such as the nonreducing colloidal disaccharide trehalose,^37^ and induction or reduction of the heat-shock protein Hsp70.^38^ The ability to survive desiccation involves a complex system of factors working at the molecular, physiological, and structural levels.^39^ An important trade is the ability of DNA damage reversion.^33,34^ Previous research in anhydrobiosis has shown that during desiccation, the cell promotes glycolysis with subsequent amino acid anabolism.^40^ This has to do with polyhydroxy compounds, mainly carbohydrates.^41^ The survival strategy is the accumulation of high concentrations of small carbohydrates, such as trehalose, sucrose, maltose, or raffinose, before drying.^42^ Trehalose is found in high concentrations in a number of anhydrobiotic organisms, including yeast, embryonic cysts of crustaceans, and nematodes.^37^ This accumulation mechanism factors into the role of specific stress proteins (e.g., late embryogenesis proteins and heat-shock proteins) allowing protection in desiccation damage.^38,41^

Many molecules were discovered, which are thought to be involved in desiccation and fluid stresses. However, the critical knowledge of the regulatory network keeping the cellular structures coherent and maintaining the metabolic machinery during dehydration is fragmentary and not well understood.^32^

The cellular mechanism of multiplication is a complex process. There may be simple microscale physical parameters that may control cellular functions, including cell division.^43^ Complete desiccation is tied with breaking of both strands of chromosomal DNA. Because of the lengthening of time in which DNA damage of animals accumulates, when in the dehydration state, a sudden need for reparation during the hydration process confirms the decline in survival after long periods of anhydrobiosis.^33^ It appears that organisms that can live through anhydrobiosis have a unique ability to fix damage acquired in the form of double-strand brakes of their DNA. It is hypothesized that this is due to sugars in a colloidal form (trehalose, sucrose, and fructose), which these animals provide for amino acid formation during and after the desiccation phase.^32^ Similar damage to cells is acquired during exposure to radiation.^44^

Survival mechanisms responsible for withstanding various environmental stresses remain largely unexplained, despite the relevance to the fast developing field of astrobiology. When we monitor these organisms (extremophiles), providing measurements of a wide range of environmental conditions resolves the specific survival strategies.^45^ When we take lowering the temperature, as an example, cellular chemistry slows down and even stops. Such an understanding of mechanism becomes one of the main goals for astrobiology.^46^

Unicellular organisms that can adapt to an extreme environment (extremophiles) are widely investigated.^47^ Little attention has been devoted to multicellular organisms. Among them, the tolerating organisms are nematodes.^48–50^ These are capable of both dehydration^51^ and containment of intracellular ice.^52^ Other two types of multicellular organisms capable of low-temperature survival are midge^53,54^ and tardigrades, capable of resistance to multiple stresses.^55^ Tardigrades became an astrobiological study model.^56,57^

## Tardigrades

Tardigrades (water-bears) are invertebrates, 0.1–1.0 mm long, and ubiquitous on Earth.^39,56,58–60^ They evolved a large variety of dormant stages that can be ascribed to diapause (encystment, cyclomorphosis, resting eggs) and cryptobiosis.^39^ Four types of cryptobiosis: anhydrobiosis (lack of water), anoxybiosis (lack of oxygen), cryobiosis (freezing), and osmobiosis (high solute concentration) is common for most.^39,40^ Tardigrades are resistant to extreme environmental changes, including desiccation, freezing, and radiation.^35,40,44,61^ In adverse environments (during cryptobiosis), they reduce their metabolic activity to undetectable levels^40,60^ and become dehydrated while forming the tun state. Such state allows them to essentially stop all bioprocesses for periods that can be a substantial portion of their life span, until environmental conditions improve and the tardigrades return to their active state.^40^

The beginning and the end phase of the cryptobiotic process is the most critical for successful survival. There are many morphological, biochemical, and physiological steps that need to be choreographed in a specific order. Therefore, the rate of desiccation and freezing, as well as rehydration and thawing, must be slow enough to provide necessary time to perform these processes without error.^58^ In addition, the time spent in the cryptobiotic state, the environmental abiotic parameters, and the conditions present during the initial and final phases (both biotic and abiotic) influence the degree of the accumulated molecular damage after a cryptobiotic event.^33,37,39,60,61^ Both high humidity and high oxygen partial pressures can have a negative impact on survival.^62^ Also, extremely low temperatures that are not available in the natural environment can prevent cryobiotic state survival.^60^ Within anhydrobiosis, problems have been reported with leakage of cell contents if anhydrobiotic animals are rehydrated directly in water without previous exposure to high humidity conditions. Direct immersion of a sample into water prevented the recovery.^62^ The continuous slow decrease in the humidity condition helps the tardigrades to dehydrate slow enough so that they can enter an anhydrobiotic state with success.^38^

When tardigrades approach dehydration (water content <3% of the hydrated animal), their body size shrinks.^44^ In their desiccation form, metabolism decreases to a nonmeasurable level and the animals survive in a dormant state until environmental conditions change (<10 years) and allow for rehydration and the resumption of normal activities.^62,63^

Entering a state of anhydrobiosis enables tardigrades to minimize or avoid severe damage to cellular structures during periods of environmental stress.^63^ One idea, which explains the maintenance of cell integrity during anhydrobiosis, is water replacement hypothesis with an important assumption about the accumulation of compatible solutes inside the cells, for example, polyhydroxy alcohols and certain nonreducing colloidal disaccharides like trehalose.^33,37,42^ They play a role in the protection of cells and biomolecules during desiccation in such a way that the loss of hydrogen bonding is compensated by reversible interactions with other molecules.^33,42^ In addition, the vitrification hypothesis proposes the formation of amorphous sugar glasses during desiccation in the cytoplasm, which protects proteins and membranes.^37,42,63^ A glass is a liquid state with solid-like properties. In a glassy state, molecules are randomly distributed, as in a liquid, but the molecular mobility is low, as in a solid state. This results in molecular immobilization of the cytoplasm, which gives protection to the dry organism.^42^

Trehalose accumulation has been detected in several species of tardigrades.^37^ However, its amount detected in them (up to 2.3% of dry weight)^37^ is significantly lower when compared with other anhydrobiotic organisms,^33,39^ and hence, trehalose accumulation does not seem to play a role in tardigrades to survive extreme desiccation.^37,39,63^ Furthermore, the nature of the stress proteins and late embryogenesis abundant proteins (found in nematodes and maturating desiccation-tolerant plant seeds) enhances our level of understanding of the tardigrade cryptobiotic mechanisms. These are connected with repairing molecular damage during rehydration.^38^

However, the anhydrobiotic ability is probably a somewhat complex set of conditions that utilizes various molecular components, like stress proteins and carbohydrates, which are choreographed together.^37,38^

In tardigrades, tolerance to low temperature is well known.^58,63^ They are considered freeze tolerant, that is, animals tolerating a limited degree of extracellular freezing.^51,58,61,63^ They survive the freezing by producing ice-nucleating agents in the extracellular fluid triggering freezing of the extracellular fluid at somewhat higher subzero temperatures. Because the process of freezing occurs at a slower rate, it allows for better control of the biochemical processes, and as a result the organism is able to withstand the damage caused by freezing. During this process, freeze-tolerant organisms are capable of cryoprotectant production.^49,58,61,63^

It has been shown that the rate at which solid-phase transition advances through the tissue has a critical influence on the tardigrades' survival. Survival of tardigrades declines with decreasing nucleation temperature and with an increasing rate of liquid/solid transition.^51,58,63,64^ Outside the cell the newly formed ice exposes the cells and connecting tissues to dehydration by freezing. This is the time when intracellular solutes start approaching saturation, while the cells osmotically dehydrate. However, when the rate of advancing the solid/liquid transition exceeds a specific limit, tardigrades experience osmotic shock, which causes mortality of unprotected cells.^58^

Tardigrades not only survive freezing in their dehydrated state but also are capable of surviving exposure to −196°C while fully hydrated.^60^ However, not too many experimental studies were concerned with the ability of tardigrades to survive freezing conditions while in a hydrated state.^60^ As much as 80% of the body water is being converted into ice providing the slow cooling rate allows for survival of several specific cryptobiotic species of tardigrades, nematodes, as well as some freeze-tolerant insects.^58^

Tardigrades are among the most desiccation- and radiation-tolerant animals and have been shown to survive extreme levels of ionizing radiation.^35,44,57,61^ They tolerate a high dose of radiation, for example, 1000s of grays of X-rays, gamma rays,^44,65^ and heavy ions.^44^

## Methods

To verify that the gas concentration in fluids of vitrified tissue control the freezing process, we conducted a controlled experiment with distilled water containing dissolved air. The water container had dimensions of 0.5×40×40 mm bounded by two glass plates superglued to a 0.5-mm-thick plastic sheet adjusted for viewing of ice formation while exposing this setup to 243 K. The freezing rate during the ice appearance was estimated as 1 K/min at the edge of the container, where the ice first appeared. Rate was estimated from the silicon diode measuring the temperature attached to the edge of the glass container. A portable camera, Gembird, operating inside the freezer, monitored the progress of freezing.

The temperature was further lowered down to 93 K, while liquid helium vapor was applied to cool the holder setup in a cryogenic Dewar with windows on both sides. In all cases, the transparent view of ice was utilized by cross-polarized light. Polarizer sheets were placed perpendicular to each other on both sides of the glass container assembly with ice. A commercial camera, Nikon Coolpix S4200, took images.

The samples used for cryopreservation were provided by Dr. Horikawa and consisted of dehydrated animals (<3% of water) deposited on a paper substrate. Animals were pure culture of Ramazzottius varieornatus^66^ that were grown on the green alga Chlorella vulgaris for food. These tardigrades' eggs require 5.7±1.1 days to hatch and animals began to deposit eggs 9 days after their hatching. Ramazzottius varieornatus has a life span of 35±16.4 days and acquired an anhydrobiotic capacity throughout the whole life cycle in the egg, juvenile, and adult stages. We took 20 of these tardigrade individuals in their dehydrated forms (tun) and 20 in hydrated forms (droplets of water) and placed them on the sample holder designed for a dilution refrigerator.

The refrigerator (dilution refrigerator with the base temperature of 0.039 K from Oxford instruments, United Kingdom) uses the Helium3 and Helium4 phase transition to achieve the low sub-Kelvin temperatures. The freezing rate for cooling between room temperature and 200 K was about 20 K/min, between 200 and 100 K was about 10 K/min, between 100 and 4 K about 2 K/min, and less than 1 K/min down to refrigerator's base temperature of 29 mK. Warming of the tardigrades happens in similar, but reversed rates only when warming between the 270 and 295 K, the rate was about 1 K/min. The testing took place for about 1 month and thus these 40 individual organisms experienced temperatures less than 1 K for that amount of time. After warming them up back to room temperature (295 K), we added a drop of water on the dehydrated and hydrated individuals and watched the signs of life under a binocular magnifier.

## Results

While rewarming Ramazzottius varieornatus^66^ from cryogenic temperature (using freezing and warming rates of 20 and 1 K/min, across the water freezing temperature, respectively) by adding a drop of water on these tardigrades, when rewarmed back to 295 K, 15 individuals (75%) showed signs of life and started to go about living. This extended cryoresistance for this species. All of the 20 hydrated individuals did not show any signs of life (movement) during the rewarming phase, when using the same conditions (freezing and warming rates of 20 and 1 K/min, across the water freezing temperature, respectively) of freezing and warming as for dehydrated individuals. Note that the freezing rates were more than two orders of magnitude faster than the rates shown to prevent survival of hydrated animals (0.1 K/min).^63^

The volume of water that was frozen completely at a temperature of 20°C first time took 18 min 17 sec. When frozen a second time, it took only 17 min 12 sec, 1 min and five seconds earlier. Each time we observed gas bubbles escaping along the ice/water interface with about less than half of the bubble numbers during the second freezing. Each time the frozen volume was larger compared with liquid water due to volumetric mismatch between the solid and liquid state of water.

When the deionized water continued to cool down to lower temperatures (Fig. 1), we observed several generations of fracturing. There were both large-scale fractures (10 mm) and small-scale fractures (0.1 mm). The large-scale fractures developed primarily at temperatures between 273 and 200 K. When cooling further toward 100 K, we registered primarily small-scale fractures.

## Discussion

We are following the developing hypothesis that the tolerance against freezing damage is improved by sugars being produced as part of the nucleus chemistry. Sugars not only are keeping the DNA strands from migration after an occurrence of double-strand break cell but also provide resources for amino acid anabolism to fix the acquired double-strand break damage after rehydration. The tardigrades' ability to repair the double-strand break damage allows for survival not only in the deep cryo-environment but also under several orders of magnitude a larger radiation exposure dose than the dose that is lethal to humans.^29,30,67^ In order to fix the DNA damage from double-strand breaks, the sugars must be either already present near the chromatin fiber, preventing the damaged strands from migrating out from their positions before breaks, thereby allowing the DNA repair mechanism to fix the double-strand brake damage.

During the cooling of tardigrades in their dehydrated state, 75% survived (15 out of 20), while 0% survived (0 out of 20) when cooled during the hydrated state. In both cases, the rate of cooling was exceeding 10 K/min. Survival of the dehydrated group indicated that during dehydration,^66^ there was enough time to start the precipitation of colloidal sugars needed for hydrating survival. When freezing the hydrated animals at the rate of 10 K/min, there was no time to allow precipitation of the cryoprotective compounds and all of the animals have died. However, there is an alternative explanation. The fast cooling rate may have rendered the precipitation of ice, which due to differential contraction acquired microfractures to cellular structures that were not possible to fix, and as a result, most of the damaged cells went through apoptosis and the animals died.

To understand this result, we ran a separate experiment when cooling the slab of water. Successful cryopreservation requires control of key physical parameters of liquids during the ice formation and an understanding of preservation processes in select natural organisms. In this case, we focus on dissolved gases in fluids. We discussed the gas content in body fluids and its effect on the formation of ice and heat conduction. We suspect that heat flow differences were present during the cooling of tardigrades in their hydrated state. Hydrated state guaranties the formation of ice connected with necessary fracturing, as observed in Figure 1, when using a cooling rate of 1 K/min. We would like to point out that fracturing is likely to be a critical parameter for tardigrades' survival in the hydrated state along with the precipitation of sugars inside the cells. Based on the evidence of fracture development (Fig. 1), we conclude that fractures represent a serious barrier for successful cryopreservation of multicellular bodies in their hydrated state. Such fracture occurrence is likely to be avoided when bodies are small enough (we detected fracture spacing smaller than 0.1 mm in Fig. 1) and the freezing rates are on the order of few degrees per hour.^63^ Therefore, even though it was suggested that the slow cooling rates are required to provide time for the metabolic and biochemical preparation of subzero exposure,^53,58,68^ we point out that, based on our data, the slow cooling rates are important from the point of view to minimize fractures in the differentially contracting solids in the small multicellular organism.

Our discussion about the reasons for freezing resistance of the selected organisms points to a need for intense dehydration of the cells before the freezing. Once the tissues are dehydrated, the absence of the surrounding volume of ice allows for the contraction of the tissue and the tissue acquires less damage. The 3% of water content may be enough to prevent not only the freezing damage but also stops all of the bioprocesses within the cells. However, dehydration only does not stop all of the bioprocesses and a cryotemperature environment may be necessary for the long-term storage of viable cryopreserved tissue.^33,34^ We propose that the problem of cryopreservation reduces prevention of cell viability during intense dehydration of cells of multicellular body, while providing the sugars for the processes connected with body revitalization. This is a necessary step to overcome the problems with both cellular fracturing and ice damage.

**FIG. 2.**
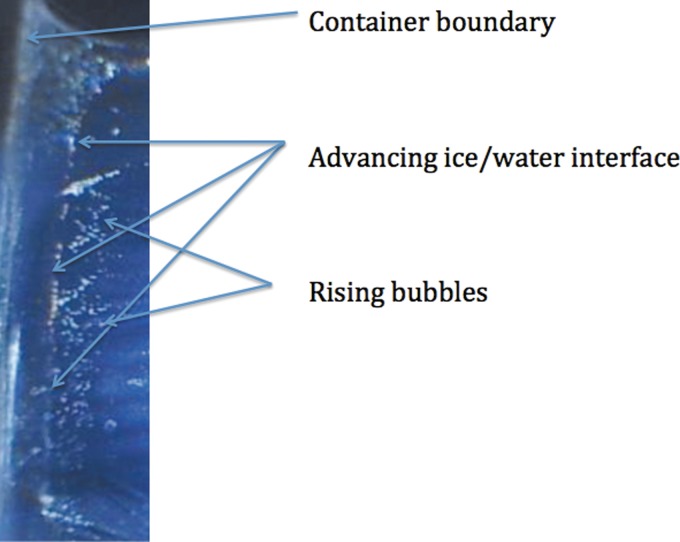
Bubbles of gas precipitate and rise near the advancing ice/water interface (from left to right). Cooling rate of the ice near the edge was 1 K/min.

In our simple experiment with a slab of fluid, the sample with larger initial concentration values of dissolved gases conducted heat at a slower rate than the gas-depleted fluid used for second freezing. Due to contrasting amounts of dissolved gases, the second material was a better heat conductor as most of the dissolved air escaped during the first freezing (Fig. 2). By analogy, when fluid contained within the cryopreserved organ transfers into the solid state, the dissolved gases are transported into the region where there are the last pockets of tissue containing liquid. These are places that contain concentrated gas solutions resulting in both aerated sugar/salt solutions and bubble formation during the transfer into the solid state. These places are the locations where the heat transfer on a microscale is more sluggish and needs to be considered as possibly compromising the homogeneous cooling by fracturing during cryopreservation attempts. Attenuation of the fractures toward the location of microbubbles in the frozen tissue was confirmed by a microscopical examination of the fractures acquired to the frozen slab (Fig. 1).

When testing the process of further cooling of a slab of water, we observed the development of fractures during cooling to temperatures near −180°C (Fig. 1). At this temperature, we have not seen much of the fracture development (except occasional small-scale fractures) when lowering the temperature beyond −180°C (−269°C), down to the temperature of liquid helium. In fact, most of the large-scale fractures developed between −60°C and −100°C (Fig. 1). We conclude that these fractures may be the critical barrier for successful cryopreservation of multicellular tissue in a hydrated state. An alternative to this explanation is that in the hydrated state, there is enough water to form intracellular ice, while in the dehydrated state, the increased relative sugar contents from the dehydration process prevent intracellular ice formation due to a lesser amount of water.

## Conclusions

Dehydrated tardigrades survive a cooling rate of 10 K/min down to 39 mK, while hydrated tardigrades do not. This is likely due to fracturing.

Fractures in frozen fluid develop preferentially at the sites of microbubbles due to dissolved gas phase prior freezing.

Fluid containing a larger amount of dissolved gases takes a longer time to transfer into a solid than the fluid containing a smaller amount of dissolved gases.

Multicellular tissue dehydration along with the tissue's exposure to colloidal sugars may be a prerequisite for successful cryopreservation of such tissue.
